# The clinical characteristics of perineal tears: A study carried out on 14 pregnant women in a tertiary center: Case series

**DOI:** 10.1016/j.amsu.2022.104432

**Published:** 2022-08-18

**Authors:** Hugues Cakwira, Marcelin Mukengere, Baraka Lucien, Abdullahi Tunde Aborode, Leonard Sironge, Meni Vhosi Michael, Aymar Akilimali

**Affiliations:** a Faculty of Medicine, Catholic University of Bukavu, Bukavu, DR, Congo; b Standing Committee of Research and Exchange, Medical Student of Association, DR, Congo; c Faculty of Medicine, Evangelic University in Africa, Bukavu, DR, Congo; d Healthy Africans Platform, Research and Development, Ibadan, Nigeria; e Faculty of Pharmacy and Public Health, Official University of Bukavu, DR Congo; f Department of Research, Research Circle, Bukavu, DR, Congo; g Faculty of Medicine, Université Libre des Pays des Grands Lacs, Goma, DR, Congo; h Faculty of Medicine, Official University of Bukavu, Bukavu, DR, Congo; i Department of Research, Oli Health Magazine Organization, Kigali, Rwanda

**Keywords:** Case series, Episiotomy, Lacerations, Perineum, Pregnancy, Trauma

## Abstract

**Background:**

The sheer quality of the female genital tract is not always respected at the time of childbirth because no protocol for the management of perineal tears exists in our services these days. The management remains dependent on a gynecologist and obstetrician. The study aimed to describe the characteristics of perineal tears.

**Methods:**

Our cross-sectional, retrospective, and descriptive study focused on patients admitted for childbirth and hospitalized in the obstetrics department of the Saint Luc Tertiary Clinic for a period from March 2021 to March 2022. During this period, we recorded 111 deliveries with 14 perineal tears.

**Results:**

A total of 111 deliveries were recorded with a 12.6% frequency of perineal tears. 64.3% of women aged between 26 and 35 and 71.4% of primiparous women were affected by perineal tears. For delivery, 64.3% of births were eutocic deliveries, with 42.9% of children born with a birth weight greater than 4 kg, and the cephalic presentation delivered 86% of children. For degrees of perineal tears, 64.3% of patients had first-degree perineal tears. For postpartum treatment of perineal tears, analgesics help calm the pain, and antibiotic therapy has been considered. For fourth and third-degree tears, episiotomy was performed as a surgical procedure.

**Conclusion:**

Perineal tears are the trauma often encountered in obstetrics; the first few suffer from it essentially. The high birth weight of children is often the cause. They require immediate management to prevent or avoid infections.

## Highlights

• Women have a very complex anatomy, hence the high risk of trauma or tears of the perineum that are often unavoidable during childbirth. Young primiparous women are the most vulnerable to perineal tears with a very high prevalence.
• The high weight and cephalic presentation of children at delivery are the factors causing perineal tears in women. Vaginal delivery and the number of foetuses has an impact on the integrity of the perineum.
• During childbirth, perineal tears should be brought to the attention of obstetric providers to avoid complications for the woman.

## 1 Introduction

During childbirth, several women may have trauma to the perineum, a large part of them come to the maternity ward to give birth for the first time [1], and perineal tears are the most common complications during pregnancy [2,3]. Based on the anatomical structures of women, perineal tears are injuries to the perineum, vulva, and vagina that occur during vaginal birth [3,4]. Risk factors for perineal tears include high birth weight or macrosomia, shoulder dystocia, instrumental deliveries, and history of perineal tears [5].

The perineal tears are grouped into four classes. The first class is the superficial lesion of the vaginal mucosa, which can also affect the skin at the level of the perineum, the second class is the tear of the first class with the vaginal mucosa and the body of the perineum, the third class is a second class tear involving the anal sphincter, and the fourth class is a third class tear involving the rectal mucosa [6,7]. Primiparity is a significant risk of perineal tears, the high weight of the child at birth (weight greater than 4 kg) or even infantile macrosomia and vaginal delivery by forceps and suction cups are also considered as risk factors [8]. Some perineal tears are treated with stitches, first-class tears do not require treatment because they do not destroy the female anatomy and are hemostatic, and other tears require an episiotomy [7,9]. Antibiotic therapy is necessary for postoperative care to avoid infection, and analgesics are also crucial in the postoperative period [7,10,11].

We carried out a retrospective and descriptive study of pregnant women who suffered perineal tears during childbirth. This work aims to describe the clinical characteristics of perineal tears.

## 2 Methods

The retrospective, descriptive and cross-sectional study was carried out over one year, the period between March 2021 and March 2022, in the department of Gynecology and obstetrics of the Clinique Tertiaire Saint Luc de Bukavu, a renowned clinic in the eastern region of the Democratic Republic of the Congo. The study is based on the documentation of patients admitted to the maternity ward for childbirth, having suffered a perineal tear and hospitalized in the gynecology-obstetrics department.

During this period, we recorded 111 deliveries, including 14 perineal tears. Patients fulfilling the inclusion criteria, including the document with complete information, whose age range is between 18 and 35 years, and those diagnosed with perineal tears were included in the study. Those whose hospital documents did not contain sufficient information for the realization of our research were eliminated or downgraded, and those not diagnosed with a perineal tear were excluded from our study. The authorization to carry out this study was granted by the authorities of the department of the Tertiary Clinic Saint Luc of Bukavu.

The results are grouped and presented in tabular form. To analyze the results, we used Microsoft Word 2016 for Windows (Version 19, Microsoft Inc.) and Microsoft Excel 2016 for Windows (Version 19, Microsoft Inc.).

This case series has been reported in line with the PROCESS 2020 guideline [12].

## 3 Results

During our study period, we recorded 111 deliveries with 14 perineal tears, including a frequency of 12.6% of tears in the gynecology and obstetrics department of the Saint Luc Tertiary Clinic in Bukavu.

In the table (Table 1), we have represented our results on the epidemiological characteristics of patients with perineal tears. Starting from the age distribution, women whose age group is between 26 and 35 years old were the most affected by perineal tears with 64.3%. The primiparous represent 71.4% of the equal distribution.

**Table 1:** The epidemiological characteristics of patients with perineal tears.

Variable	Effective (%)
***Distribution of age for women at childbirth*** 18–25 years 26–35 years	5 (35.7) 9 (64.3)
***Distribution of parity*** Primiparous Multiparous	10 (71.4) 4 (28.6)

The clinical features of perineal tears are shown in the table. (Table 2), we described information on the mode of delivery where we found 64.3% of eutocic deliveries. For the weight of the child at birth, which we evaluated in kilograms, we found that the majority of children were born weighing more than 4.3 kg, including 42.9%. For the child's presentation at birth, 86% of children were taken by the cephalic presentation. 85.7% of women carried a single fetus in their womb.

**Table 2:** The clinical characteristics of patients admitted for perineal tears.

Variable (total 14)	(%)
***Childbirth type*** Eutocia Dystocia	64.3 35.7
***The weight of the child at birth*** 2.5–3.5 kgs. 3.5–4.3 kgs. Upper to 4.3 kgs.	21.4 35.7 42.9
***Presentation of the child at birth*** Cephalic Seat presentation Cross-sectional presentation	86 7 7
***Number of fetus in the uterus*** 1 Upper to 1	12 (85.7) 2 (14.3)

Kgs. (kilo grams) and % (percent).

Regarding the distribution of the age of pregnancy that we evaluated in the week of amenorrhea, we found that the age is between 37 and 41 weeks of amenorrhea which occupies the most significant part with a percentage of 64.3 follow-ups of the age group more critical than 42 weeks of amenorrhea with 35.7%. Regarding the degrees of perineal tears, 64.3% of patients in our study had first-degree perineal tears, followed by those with a fourth-degree tear.

Concerning the postpartum treatment of perineal tears in the gynecology and obstetrics department of the Clinique Tertiaire Saint Luc, all the women had analgesics to calm the pain. They all received antibiotics for the prophylactic treatment of postoperative infections. Local anesthesia was given to all women before receiving stitches on the torn part. Those with fourth and third-degree tears received an episiotomy as surgical treatment.

## 4 Discussion

In the study, we find a somewhat high prevalence of perineal tears compared to childbirth. The primiparity and the age of the mother being factors of the perineal tears, we found that the primiparous whose age is between 26 and 35 years were victims of the tears; thus, the multiparity is less exposed to the risk of tears about primiparity. The number of fetuses has a significant impact on the integrity of the perineum, and there is a high number of perineal lesions in women who have delivered more than one fetus vaginally. Increased weight and cephalic presentation are risk factors for perineal tears caused by the fetus. First-degree and fourth-degree perineal tears were primarily recorded in our study.

In our region, we have noted a significant and increasing risk of perineal tears. The frequency of perineal tears is somewhat high in the gynecology and obstetrics departments. Numerous studies demonstrate the same results. These authors carried out respectively a prospective observational study during 4 months with a frequency of 12.6% and a descriptive, cross sectional and retrospective study during a period of one year [13,14].

The high frequency of perineal tears in primiparous women in our study would be linked to a lack of follow-up during the labor period, the lack of experience on the measures to be considered during childbirth, and the preventive measures to preserve the perineum. Other young primiparous women come to the ward just a few hours before giving birth when they did not attend the antenatal consultations [2]. As other authors show us, perineal tears are primarily found in young women, and these women are often primiparous. The author *Álvarez-González M* et al., shows us in her experimental study on perineal trauma that young women in their thirties are the most affected and vulnerable to perineal tears with a very high frequency [15]. The high prevalence of tears in our region shows good monitoring of young women during childbirth and the implementation of preventive measures to protect the perineum. The significant link between the qualification of the service provider and the occurrence of a tear in the perineum was not taken into account by our study since no association was found between these two variables.

In multiparous women, they are less exposed to the risk of perineal tears compared to primiparous women. This is explained by the fact that a multipara masters the measures to be considered during pregnancy, and she has a good experience with childbirth. Multiparous women have reasonable control of their perineum. They can feel when a push is effective and how to carry out their pushing technique with the help of nursing staff, unlike primiparous women who also carry out a long stimulant which alters the distension capacity of the perineum [16,17].

The high prevalence of tears in our region reflects good surveillance of young women during delivery and the implementation of preventive measures to protect the perineum. The significant link between the provider's qualification and the occurrence of a perineal tear was not taken into account by our study since no association was found between these two variables. The mode of delivery is an essential element to consider in cases of perineal tears. In our study, we recorded that a high frequency of perineal tears is observed during eutocic deliveries due to the increased weight of children during delivery and cephalic presentation [5].

Children whose birth weight is more significant than 3500 g significantly increase the risk of perineal tearing, and macrosomia is the cause that leads to mechanical dystocia. *D'Souza JC.* et al. found and demonstrated the same results [8]. The fetal presentation also plays a vital role in the occurrence of perineal lesions (tears in the perineum) when the child's weight is very high compared to normal and transverse presentations often lead to obstructed deliveries, which also give their turns cause perineal tears [5]. This explains why the nursing staff must take all the necessary measures to minimize the complications that may arise in the event of dystocia during childbirth and to reduce the risk of tearing the perineum. *Cahill A* et al. noted that nulliparous women receiving neuraxial anesthesia during the second stage of pushing efforts did not affect the rate of spontaneous vaginal delivery [18].

Multiparous women have reasonable control of their perineum. They can sense when a push is effective and how to perform their pushing technique with the help of a nurse, unlike primiparous women who also perform a long push that impairs the ability to distend the perineum. *Smith* L*.A* et al. also found that many multiparous women delivered vaginally with intact perineum compared to fewer primiparous women [13].

The mode of delivery is an essential consideration in cases of perineal tears. We have observed that infants whose birth weight was more significant than 3500 g enormously increase the risk of perineal tears, and macrosomia is the cause that leads to mechanical dystocia, The authors *Marschalek ML* et al.*,* and *Al Ghamdi DS* demonstrated the same results in their retrospective studies over a period of 6 and 5 years respectively with a mean birth weight of 4800 g presented at delivery [19,20].

The presentation of the fetus also plays an important role; we have observed a significant number of perineal tears in women with posterior occipital delivery, which corresponds to what we find in *Hauck YL* et al. [21]. The occurrence of perineal lesions (perineal tears) was more critical when the child's weight was more significant than average or in transverse presentations by complicated dystocic deliveries. This explains why caregivers must take all necessary measures to minimize the complications that may arise from dystocia during delivery and to reduce the risk of perineal tears [19].

During our study, carried out in a tertiary treatment center, a center respecting the guidelines of an international hospital. We have found that few women suffer from perineal tears during childbirth, these tears are mostly first degree and fourth degree. We have found that young and primiparous women are the most affected by perineal tears. The high weight of children and the cephalic presentation of children during childbirth increase the risk of perineal tears.

We recorded the following limitations in our study: the lack of necessary information on the patient charts, the non-availability of laboratory results on the charts and the lack of specific impacts on the weight and height of the patients. Our study comes up against the fact that many women do not attend tertiary health facilities for better care during childbirth because of the fees that are required, hence a low number of recorded cases.

### 4.1 Recommendations

To reduce the incidence of perineal tears, the medical team must consider the various risk factors that pregnant women present before delivery and propose a more favorable outcome. The obstetrician should consider the option of the cesarean section instead of mechanical obstructed labor on the pelvis, especially in a high birth weight fetus in a young primiparous mother. The birthing team should increase surveillance in women with two or more risk factors during labor to decide the outcome of birth in advance and with a high degree of flexibility to avoid perineal tears.

## 5 Conclusion

This present work has as a study of the clinical characteristics of perineal tears in women. The female anatomy being very complex, perineal tears are anatomically lesions in the perineum, vulva, and vagina that occur during vaginal childbirth. These tears are classified into four whose treatment is surgical (episiotomy) accompanied by antibiotics and analgesics. From this study, we concluded that primiparity and young age of the mother are factors increasing the risk of perineal tears and high weight as well as cephalic presentation in the child at birth.

## Ethical approval

This study protocol was approved by the Ethics Review Board of the Faculty of Medicine, Official University of Bukavu.

## Sources of funding

This research did not receive any specific grant from funding a commercial, or not for profit sectors.

## Author contribution

Conceptualization: Aymar AKILIMALI. Methodology: Hugues CAKWIRA. Software: Aymar AKILIMALI. Validation: Marcelin MUKENGERE. Formal analysis: Marcelin MUKENGERE, MENI VHOSI Michael. Investigation: Aymar AKILIMALI, BARAKA Lucien, Leonard SIRONGE. Resources: Marcelin MUKENGERE. Data curation: Marcelin MUKENGERE. Writing – Original Draft: Aymar AKILIMALI, Hugues CAKWIRA. Writing – Review & Editing: Aymar AKILIMALI, Hugues CAKWIRA, Abdullahi TUNDE ABORODE. Visualization: Abdullahi TUNDE ABORODE. Supervision: Hugues CAKWIRA. Project administration: Aymar AKILIMALI. Funding acquisition: Marcelin MUKENGERE.

## Registration of research studies

1. Name of the registry: **aymar akilimali**
2. Unique Identifying number or registration ID: **researchregistry8085**
3. Hyperlink to your specific registration (must be publicly accessible and will be checked): https://www.researchregistry.com.browse-the-registry#home/

## Guarantor

Aymar AKILIMALI is the guarantor of this study and accept full responsibility for the work and the conduct of the study, has access to the data and controlled the decision to publish.

## Consent

The information used in this study does not include any identifying information from the patients; thus, written consent from the patients was not required.

## Data availability

Not applicable.

## Declaration of competing interest

The authors do not report any conflict of interest.
